# LASCA: loop and significant contact annotation pipeline

**DOI:** 10.1038/s41598-021-85970-4

**Published:** 2021-03-18

**Authors:** Artem V. Luzhin, Arkadiy K. Golov, Alexey A. Gavrilov, Artem K. Velichko, Sergey V. Ulianov, Sergey V. Razin, Omar L. Kantidze

**Affiliations:** 1 Institute of Gene Biology Russian Academy of Science, Moscow, Russia; 2 Center for Precision Genome Editing and Genetic Technologies for Biomedicine, Institute of Gene Biology Russian Academy of Sciences, Moscow, Russia; 3 Institute for Translational Medicine and Biotechnology, Sechenov First Moscow State Medical University, Moscow, Russia

**Keywords:** Genome, Bioinformatics, Chromosomes

## Abstract

Chromatin loops represent one of the major levels of hierarchical folding of the genome. Although the situation is evolving, current methods have various difficulties with the accurate mapping of loops even in mammalian Hi-C data, and most of them fail to identify chromatin loops in animal species with substantially different genome architecture. This paper presents the loop and significant contact annotation (LASCA) pipeline, which uses Weibull distribution-based modeling to effectively identify loops and enhancer–promoter interactions in Hi-C data from evolutionarily distant species: from yeast and worms to mammals. Available at: https://github.com/ArtemLuzhin/LASCA_pipeline.

## Introduction

Techniques exploiting the proximity ligation procedure (so-called C-methods) have significantly improved our understanding of the spatial (3D) genome organization. C-methods have confirmed the existence of chromosomal territories that are spatially compartmentalized into active and repressed chromatin domains, referred to as A and B compartments^1^. These megabase-scale compartments are partitioned into self-interacting structures, termed topologically associating domains (TADs). The distinguishing feature of TADs is that spatial contacts of remote genomic elements are more frequent within TADs than between individual TADs^2–4^. Boundaries between individual TADs are enriched with cohesin complex and CCCTC-binding factor (CTCF). Recent data show that TADs are formed via dynamic DNA loop extrusion and may harbor smaller contact domains, some of which are chromatin loops^5,6^. Along with chromatin compartments and TADs, chromatin loops represent one of the major levels of hierarchical folding of the genome. Although most of the loops in mammals are anchored by CTCF and cohesin, several other proteins can mediate long-distance genomic interactions: Yin Yang 1 (YY1), zinc finger protein 143 (ZNF143), LIM domain-binding factor 1 (LDB1)^7^. All the three are involved in establishing enhancer-promoter interactions either by direct binding and bridging specific genomic sites (YY1 and ZNF143)^8,9^, or by binding to a subset of transcription factors (LDB1)^10^. Species that lack CTCF-dependent loops can however utilize DNA extruding complexes such as condensin and cohesin to organize their genomes or genome parts into consecutive similar-sized chromatin loops. Specifically, *Caenorhabditis elegans* X chromosome contains dozens of loops associated with a condensin-like dosage compensation complex (DCC)^11,12^. In budding yeast, S-phase chromatin forms consecutive loops which base points often colocalize with binding sites of cohesin protein Scc1^13^.

In contrast to chromatin compartments and TADs, chromatin loops have clear biological roles, such as bringing gene promoters to their cognate *cis*-regulatory elements^14^. Hence, the identification of loops is an essential part of most studies that involve Hi-C-based 3D genome analyses. Methods that have been developed to detect chromatin loops and/or statistically significant genomic interactions from Hi-C contact maps comprise two groups: (1) statistical/probabilistic model-based methods (e.g. Fit-Hi-C^15^, HiC-DC^16^), and (2) peak-calling methods (e.g. HiCCUPS^17^, MUSTACHE^18^, SIP^11^, cLoops^19^). All of them are primarily focused on mapping the loops in Hi-C datasets from mammals, and experience some (in most cases insurmountable) challenges working with Hi-C datasets from animal species with substantially different genome architecture. Here, we present the LASCA pipeline that uses Weibull distribution-based modeling to effectively identify chromatin loops, including enhancer-promoter interactions, in Hi-C data from different animal species (human, mouse, nematode, budding yeast). Our results demonstrate that LASCA-detected loops are (1) reproducible, (2) highly supported by aggregate peak analyses and genomic/epigenomic correlates of loop formation, (3) validated by protein-centric chromatin conformation methods (ChIA-PET and HiChIP). We have compared LASCA with the most commonly used methods from each of the abovementioned groups (HiCCUPS and Fit-Hi-C) and with a very recent approach MUSTACHE. Working with mammalian Hi-C data, LASCA showed very similar results to HiCCUPS and MUSTACHE, and even outperformed HiCCUPS in detecting CTCF-independent loops. In contrast to methods compared, LASCA could also detect chromatin loops in *C. elegans* and *S. cerevisiae*, which makes it an omni-purpose approach.

## Results and discussion

### Description of the LASCA pipeline

**Figure 1:**
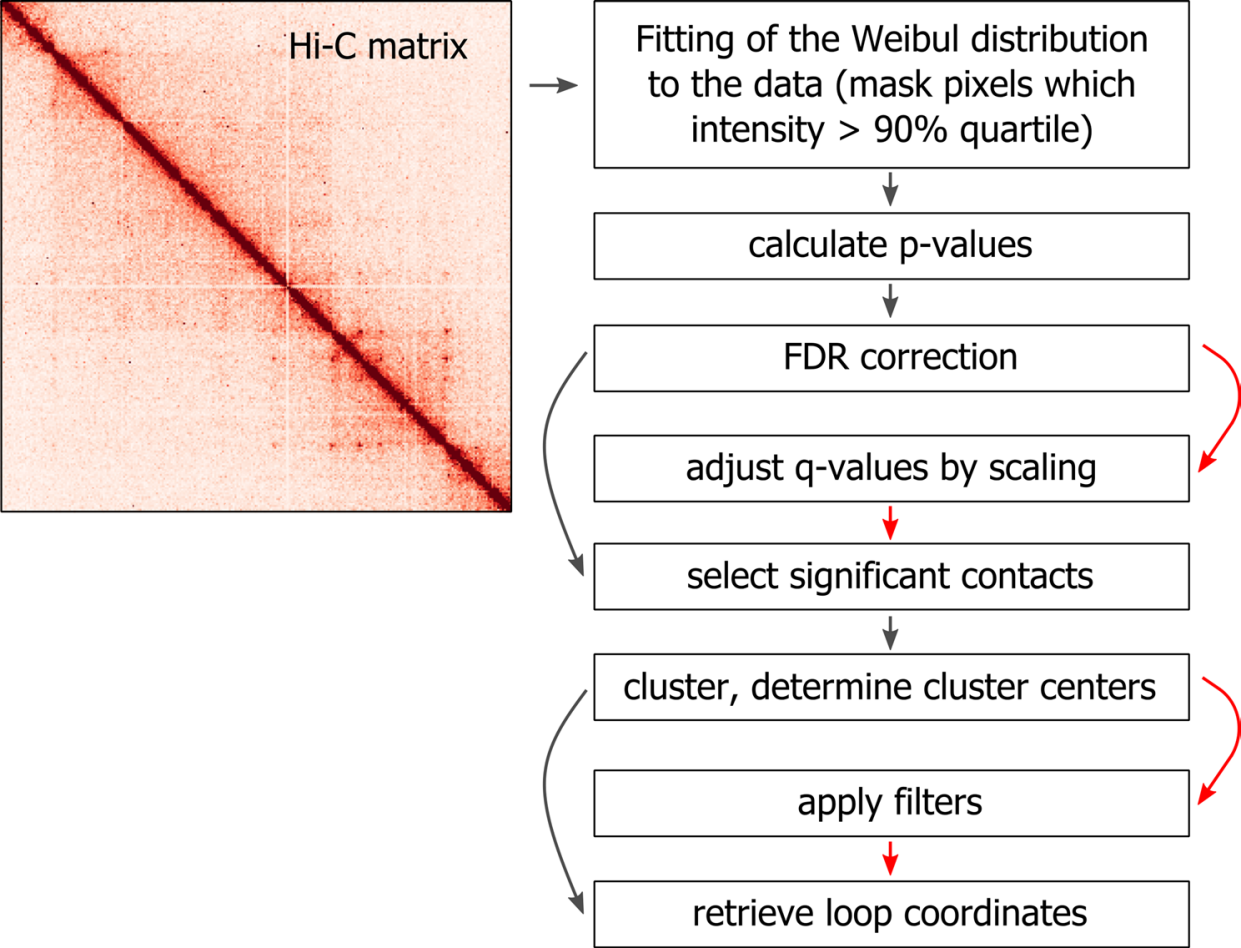
Overview of the LASCA pipeline. Optional steps are marked by red arrows.

In Hi-C heatmaps, loops appear as bright dots located at different distances from a central diagonal. Here, we applied Weibull distribution-based modeling^20–22^ to identify these significant interactions. Comparison of several statistical distributions (Weibull, normal, log-normal, and gamma) performed in Sanyal et al.^20^ clearly showed that the Weibull distribution fits Hi-C interaction frequency the best. We further analyzed the quality of the Weibull distribution fitting of Hi-C interaction frequency at different distance ranges and found that its good performance is distance-independent (Supplementary Fig. S1). The LASCA pipeline works with corrected Hi-C matrices in a Cooler format^23^. First, the Weibull distribution-based statistical background model is fitted to each diagonal of the Hi-C matrix (Fig. 1). The p-value for every pixel in the heatmap is calculated as the probability of finding a corresponding model pixel with the same or higher intensity. To obtain corrected *p*-values—q-values, the Benjamini–Hochberg method is applied. Optionally, q-values are additionally corrected in accordance with the scaling of a particular chromosome. A q-value threshold level is defined by a user to determine significant pixels (contacts). Adjacent significant pixels are grouped into clusters; for each cluster, the center is defined, and its coordinates are retrieved and considered as the loop coordinates. Identified loops may be filtered according to their aggregate peak analysis (APA)^17^ or peak analysis (PA)^11^ scores, signal intensity, and signal enrichment over random signals located at the same distance from the central diagonal. The loops should also display a signal decay from the central loop pixel. These filters are optional and should be used depending on the animal species under study. Specifically, using these filters with mammalian Hi-C data will enhance the accuracy of loop annotation, whereas utilizing the filters with the yeast genome appears to be less useful.

### Identification of chromatin loops with the LASCA pipeline

**Figure 2:**
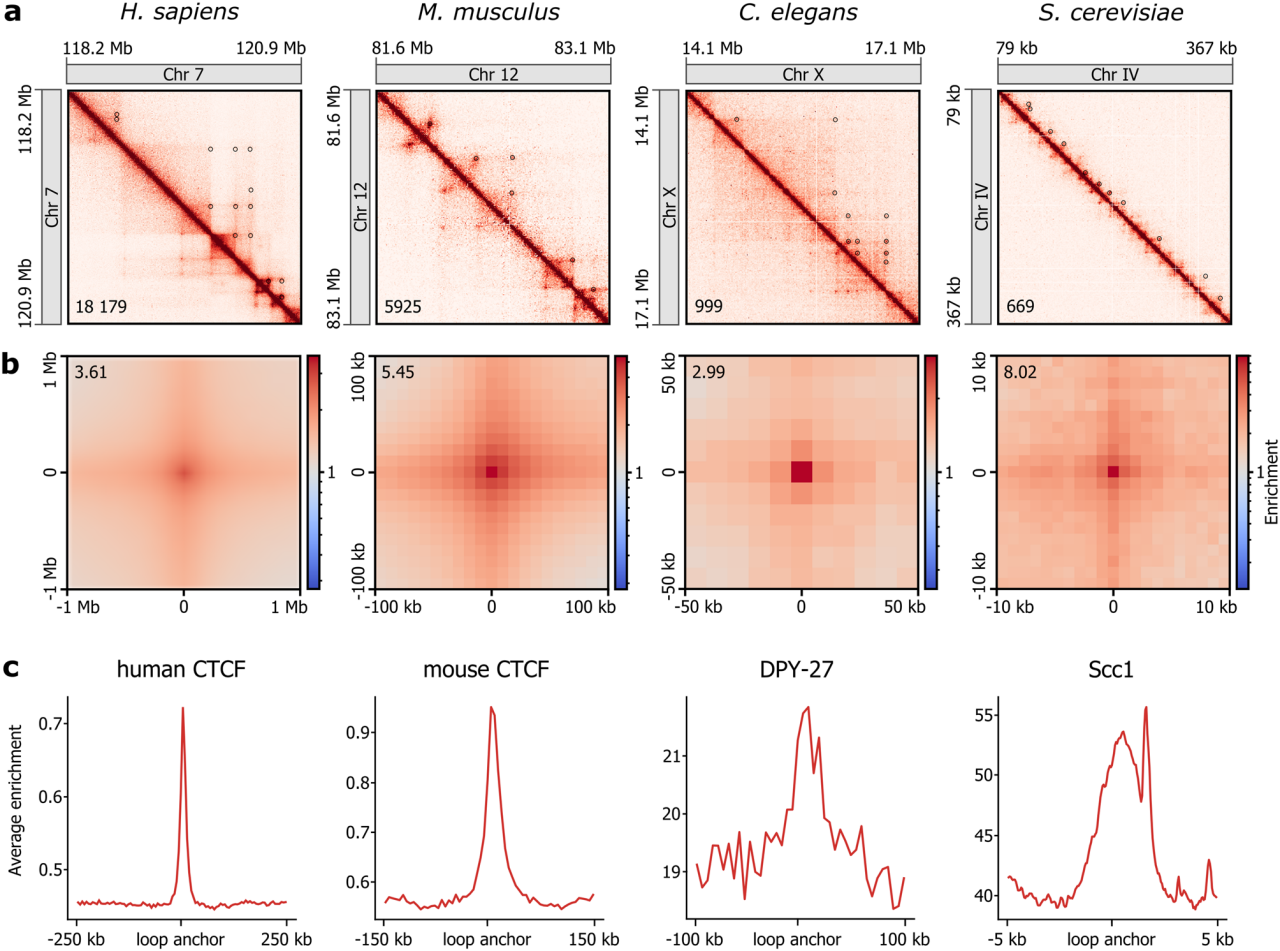
LASCA pipeline can be used to detect loops in different species. (**a**) Example loci for LASCA loops mapped in Hi-C datasets of *H. sapiens*, *M. musculus*, *C. elegans*, and *S. cerevisiae*. A number in the lower left corner of the heatmap shows the total number of LASCA-detected loops in each case. (**b**) Average loops identified by LASCA in *H. sapiens*, *M. musculus*, *C. elegans*, and *S. cerevisiae* Hi-C datasets. A number in the upper left corner shows the enrichment of contacts inside the loop pixel over the background. (**c**) Enrichment of ChIP-seq-obtained binding sites of the corresponding chromatin architectural proteins of *H. sapiens, M. musculus, C. elegans*, and *S. cerevisiae* around the anchors of the loops detected by LASCA.

We applied the LASCA pipeline to identify chromatin loops in Hi-C data from evolutionarily distant species: *H. sapien*s (GM12878 cells^17^), *M. musculus* (CH12.LX cells^17^), *C. elegans*^24^, and *S. cerevisiae* (S-phase cells^13^). We managed to annotate a significant number of loops in each case (Fig. 2a). Visual inspection of loop-annotated Hi-C heatmaps suggested the good accuracy of the LASCA in loop identification, particularly in worm and yeast datasets (Fig. 1a). We further evaluated the quality of loop mapping using a widely accepted metaplot analysis^17,25^ (Fig. 2b). Loops in human and mouse datasets displayed a classic decayed signal from the loop center to surrounding regions with a crosshair pattern that corresponded to loop extrusion^11^. Similar though less saturated patterns were observed for worm and yeast loops (Fig. 2b). We also showed that anchors of loops identified by LASCA were enriched in ChIP-seq mapped binding sites of proteins important for looping: human or mouse CTCF, condensin subunit DPY-27 of *C. elegans*, and yeast cohesin subunit Scc1 (Fig. 2c). Approximately 60% of the LASCA-detected loops showed the enrichment by the corresponding chromatin architecture factor at least at one of their base points (hCTCF—64%; mCTCF—56%; DPY-27—76% for X chromosome; Scc1—57%). Together, these results demonstrate the good performance and versatility of the LASCA pipeline.

**Figure 3:**
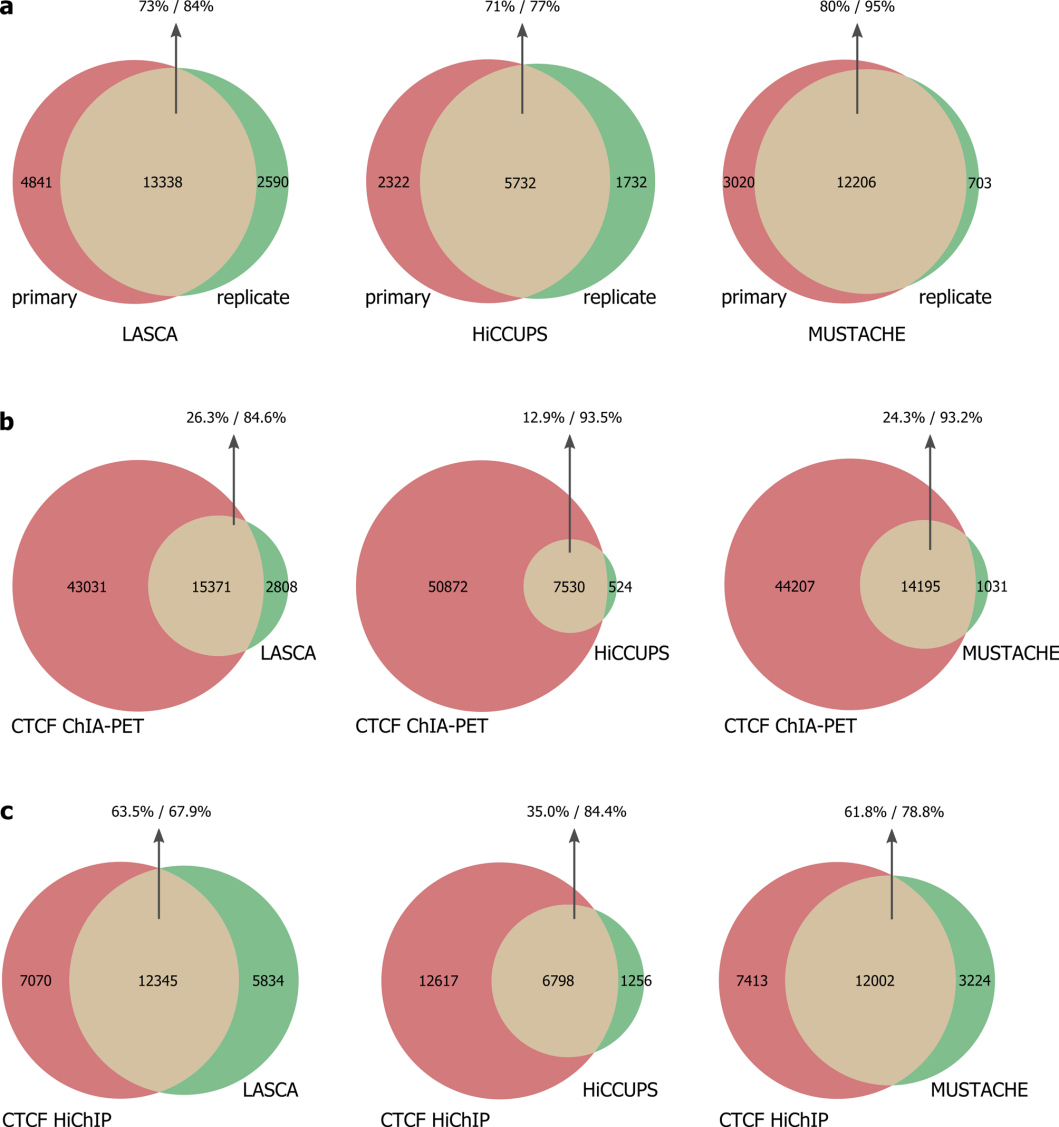
Reproducibility and accuracy of LASCA pipeline. (**a**) The overlap between loops identified by either LASCA, MUSTACHE or HiCCUPS in two Hi-C replicates of GM12878 cell line. (**b–c**) The overlap between either LASCA, MUSTACHE or HiCCUPS loops in GM12878 cells and loops detected by CTCF ChIA-PET (**b**) or by CTCF HiChIP (**c**). The overlap between the two loop sets is shown in yellow, and the percentages of overlap with respect to each set are reported separately.

To assess the reproducibility of LASCA loop calls we compared its performance on two replicates of GM12878 cell line Hi-C matrices. The results indicate that LASCA has a very high self-consistency level (73.4%/83.7%; Fig. 3a) which is comparable or even better than those of HiCCUPS and MUSTACHE (Fig. 3a). To validate LASCA-predicted loops, we compared them with loops predicted in GM12878 cells by protein-centric C-methods, CTCF ChIA-PET^22^ and CTCF HiChIP^26^. The vast majority of the chromatin loops detected by LASCA were recapitulated by ChIA-PET (84.6%) and HiChIP (68%) loops (Fig. 3b, c). Virtually the same performance in this analysis showed MUSTACHE and HiCCUPS (Fig. 3b, c). Taken together these results confirm the high accuracy and reproducibility of the LASCA pipeline.

**Figure 4:**
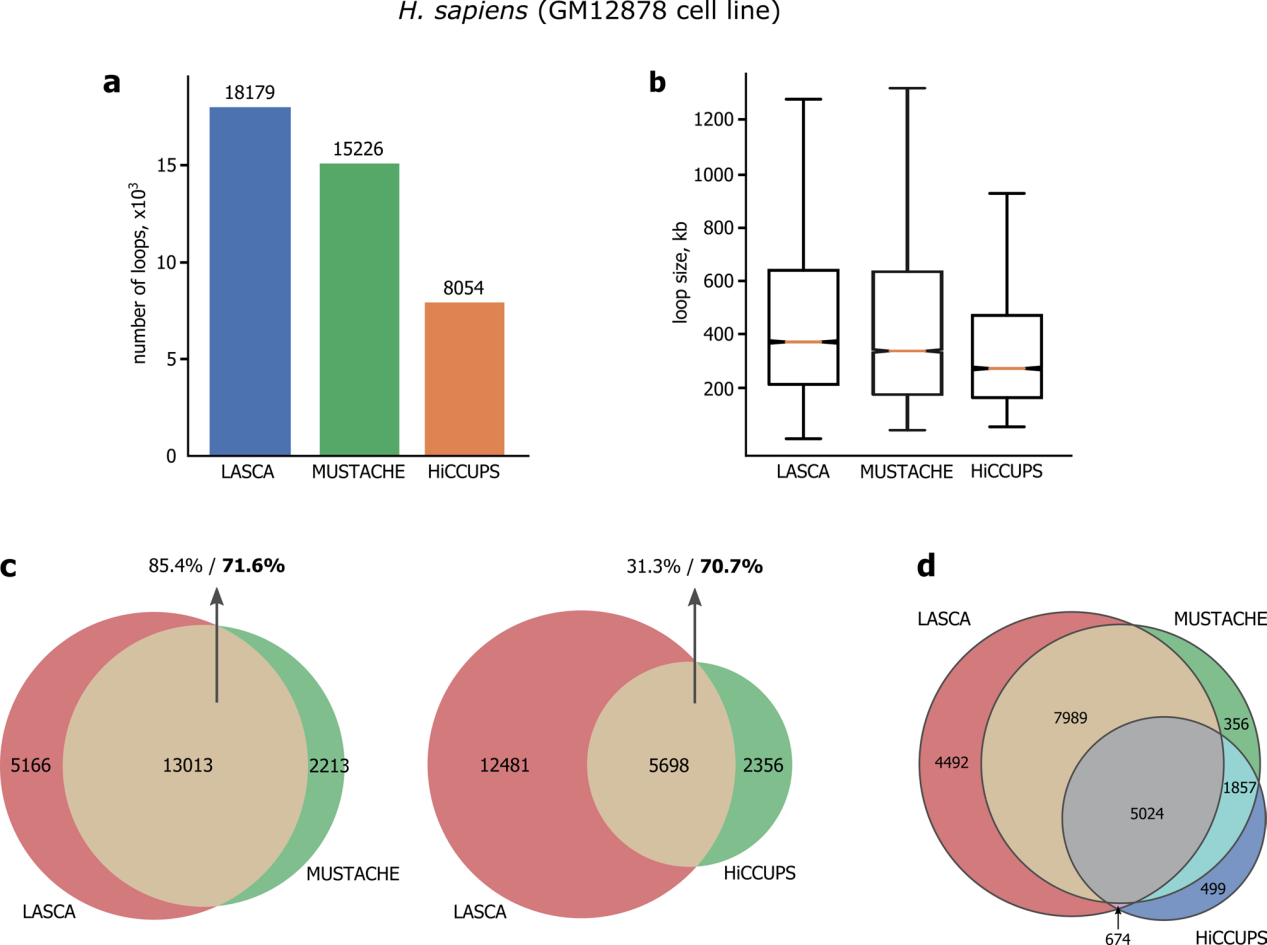
Comparison of LASCA pipeline with HiCCUPS and MUSTACHE. (**a**) Number of loops identified by LASCA, HiCCUPS and MUSTACHE in human Hi-C datasets (GM12878 cell line). (**b**) Sizes of loops identified by LASCA, HiCCUPS and MUSTACHE in GM12878 cells. Horizontal lines represent the median; upper and lower ends of boxplot show the upper and lower quartiles, the whiskers indicate the upper and lower fences. (**c**) The agreement between LASCA and MUSTACHE, and LASCA and HiCCUPS loops is shown as Venn diagrams. The overlap between the two loop sets is shown in yellow, and the percentages of overlap with respect to each set are reported separately. (**d**) Triple Venn diagram showing the intersection of loop sets identified by LASCA, MUSTACHE and HiCCUPS.

We compared the performance of LASCA with the most commonly used chromatin loop- and significant contact-detecting methods, HiCCUPS and Fit-Hi-C, and with a very recent approach MUSTACHE. It was not possible to adequately compare LASCA with Fit-Hi-C because the latter identified several orders of magnitude more contacts as compared to LASCA (~ 48 million in GM12878 cell line and ~ 34.5 thousand in *S. cerevisiae*). Nevertheless, we found that most of the LASCA-identified chromatin loops belonged to significant genomic contacts detected by Fit-Hi-C (97.3% of GM12878 loops, and 94.6% of yeast loops). LASCA identified approximately the same number of loops as MUSTACHE and two times more loops than HiCCUPS in Hi-C datasets from both human and mouse (Fig. 4a and Supplementary Fig. S2). Loops identified by LASCA had a larger median size as compared to HiCCUPS-called loops (Fig. 4b). Direct comparison of loops called by LASCA, HiCCUPS, and MUSTACHE demonstrated a good overlap (Fig. 4c). Approximately 70% of HiCCUPS loops and 85% of MUSTACHE loops in GM12878 cells were identified by LASCA (Fig. 4c).

It is noteworthy that a significant portion of LASCA loops (60%) was not mapped by HiCCUPS (Fig. 4c). To find out the characteristics of these additional LASCA-identified loops in human cells, we performed metaplot analysis and checked the presence of CTCF binding sites and specific epigenetic features (ATAC-seq and histone H3K27Ac peaks) at the bases of i) loops identified by both LASCA and HiCCUPS, ii) HiCCUPS-specific loops, and iii) LASCA-specific loops (Supplementary Fig. S3). The results obtained clearly showed that HiCCUPS detected mostly CTCF-dependent loops with a high proportion of active enhancers marked by H3K27 acetylation (Supplementary Fig. S3). LASCA-specific loops, at the same time, were slightly different in structure as evidenced from metaplot analysis, did not strongly depend on CTCF, and were not associated with the active enhancers (Supplementary Fig. S3). In summary, these results suggest that LASCA identifies additional loops that are not annotated by HiCCUPS.

### Identification of enhancer-promoter interactions with the LASCA pipeline

**Figure 5:**
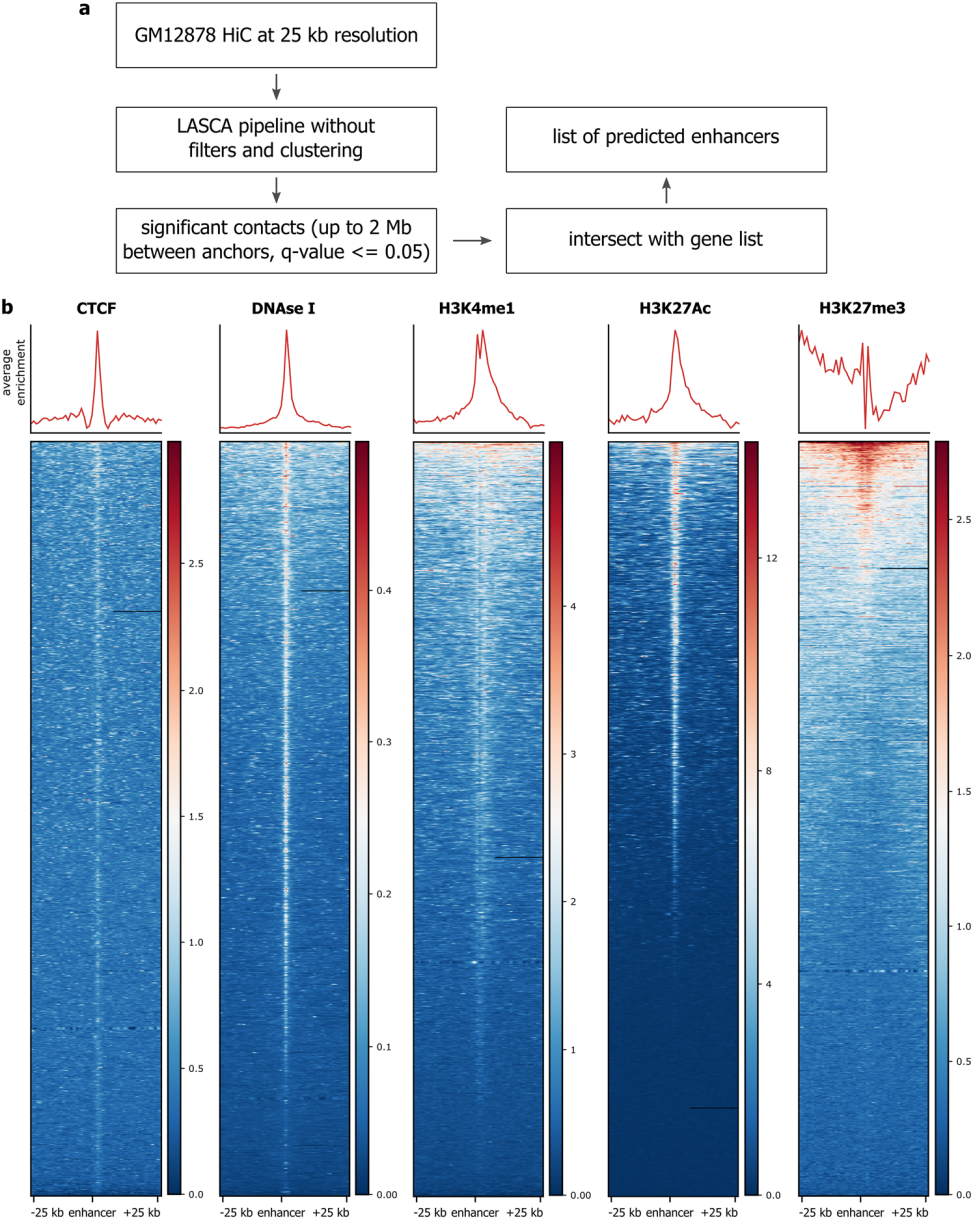
LASCA pipeline is suitable for identification of enhancer-promoter interactions. (**a**) Scheme showing enhancer-promoter interaction mapping using LASCA pipeline. (**b**) Epigenetic profiles around enhancers (+ / − 25 kb) identified by LASCA pipeline.

The LASCA pipeline is suitable for the identification of enhancer-promoter interactions (Fig. 5a). In this case, LASCA is run without chromatin loop-specific filters and retrieves coordinates of all significant genomic contacts on distances of up to two megabases. Anchors of the contacts are then intersected with a list of gene promoters; the contacts (loops) with one of the two anchors coinciding with a promoter are selected for further analysis. In these loops, the second anchor is considered to be an enhancer. To verify that the LASCA did annotate enhancers, we tested the epigenetic profile around the predicted enhancers. This profiling clearly illustrated that regions that were predicted to be enhancers displayed typical enhancer-specific epigenetic marks (Fig. 5b).

## Conclusions

LASCA allows mapping of chromatin loops as well as enhancer–promoter interaction in Hi-C datasets obtained from animal species with different genome size and genome organization complexity, such as human, mouse, worm, and yeast. To annotate representative loops/contacts, LASCA requires minimal adjustments and filtering, particularly when analyzing worm or yeast data. High-quality performance with Hi-C data from *C. elegans* and *S. cerevisiae* distinguishes LASCA from most other chromatin loop callers. LASCA, the protocol, and suite of scripts, are publicly available at https://github.com/ArtemLuzhin/LASCA_pipeline.

## Methods

### Loop annotation

The LASCA pipeline consists of three main steps (Fig. 1). In the first step, the Weibull distribution-based statistical background model is fitted to each diagonal of the corrected Hi-C matrix; for each pixel, *p*-values are calculated as the probability of finding a model pixel with the same or higher intensity, and FDR correction of the p-values is performed to obtain corresponding q-values (default argument: 0.1). Additionally, q-values may be corrected in accordance with the scaling of a particular chromosome (default argument: turned off). Briefly, for the selected range of diagonals of the Hi-C matrix, the average value of the contact frequency for each diagonal is calculated. Then, all values in the obtained set of average values of the contact frequencies are divided by the average value of the contact frequency in the first diagonal, thus forming a set of normalization coefficients with a value = 1 in the first diagonal. The q-values in each diagonal are divided by the corresponding normalization coefficient determined for this particular diagonal. The q-value threshold is defined by a user (default argument: 0.1) to determine significant pixels (contacts).

On the second step, adjacent significant pixels are grouped into clusters using a density-based spatial clustering of applications with noise (DBSCAN) algorithm (default argument: minimum cluster size is three pixels). Pixels are assigned as neighbors related to a particular cluster if the maximum Euclidean distance between these two pixels = 1. The size of the cluster (in pixels) may be specified by a user. For each cluster, the center is defined (default argument: the brightest pixel), and its coordinates are retrieved and considered as the loop coordinates. The cluster center is defined as either the arithmetic mean of the x and y coordinates of pixels in a cluster or a pixel in a cluster possessing maximum intensity (default option).

On the third step, identified loops may be subjected to various filters, such as the enrichment of signal over background (APA and PA scores (default arguments: >  = 1.9 and >  = 1, correspondingly), signal intensity, signal decay from the center of a cluster, and signal enrichment over random signals at the same distance. All steps and parameters in the LASCA pipeline can be turned on/off and adjusted by the user (default argument: turned off).

APA-score^17^ was calculated as the ratio of the intensity of the central pixel of the loop to the average intensity of the right corner of the 11 × 11 pixel-circle around the center of the loop. PA-score^11^ was calculated as the average ratio between the nearest pixels to the center of the loop and the average intensity of the right corner of the 11 × 11 pixel-circle around the center of the loop.

Using the LASCA pipeline, we identified loops in several organisms. For human cell line GM12878 Hi-C maps, loops were annotated at 5 and 10 kb resolution followed by merging of overlapped loops and filtering them by signal enrichment over random signals located at the same distance. The following parameters were used for 5 and 10 kb Hi-C maps: q = 0.95, adjust_by_scale = True, q_value_trhd = 0.2, scaling_q_value_trhd = 0.2, min_cluster = 2, filter_zeros = 2, filter_PA = 1, filter_APA = 1.9, filter_intensity = 0.3. For mouse cell line CH-12LX, loops were identified at 10 kb resolution with the following parameters: q = 0.95, adjust_by_scale = True, q_value_trhd = 0.1, scaling_q_value_trhd = 0.05, min_cluster = 3, filter_zeros = 2, filter_PA = 1, filter_APA = 1.8, filter_intensity = 0.1. For *C. elegans*, loops were annotated at 5 and 10 kb resolution with a subsequent merging of overlapped loops. The following parameters were used: q = 0.95, adjust_by_scale = False, q_value_trhd = 0.05, min_cluster = 2, filter_zeros = 2, filter_PA = 1, filter_APA = 1.7, filter_intensity = 0.1. Finally, for *S. cerevisiae*, loops were mapped with following settings: q = 0.95, adjust_by_scale = False, q_value_trhd = 0.01, min_cluster = 3, filter_zeros = 2, filter_intensity = 0.1.

HiCCUPS loops for GM12878 (primary) and CH12-LX cells were obtained from Rao et al.^17^ (GSE63525). To identify loops in GM12878 cells using MUSTACHE, we annotated the loops separately on the 5 and 10 kb maps with the following parameters: -pt 0.05, -st 0.88, -sz 1.6, -oc 2, -i 10. Then we merged the loop lists found at different resolutions. Significant contacts in GM12878 cells and *S. cerevisiae* were also annotated using FitHiC2 with default settings, except for the -U option (2 Mb and 40 kb, respectively). We cut off significant contacts by q <  = 10^−9^.

We used Bedtools to count the overlapping of the loops. We considered the loops overlapped if at least 70% of the reciprocal intersection of regions between loop base points was observed.

### Metaplot and enrichment analyses

We constructed metaplots for each of the selected organisms using Coolpup.py^25^ with default settings, except –pad parameter, which was 10 for *S. cerevisiae* and 50 for *C. elegans*.

To analyze the enrichment of proteins involved in looping, we applied deepTools2^27^ with—reference Point TSS (upstream loop anchor), and -a and -b parameters were selected as half of the mean loop size for each Hi-C dataset. In the case of enhancer-promoter interaction analysis, we set -a and -b parameters to 2 Mb.

### ChIA-PET and HiChIP loop identification

A table containing the contacts of the CTCF-PET clusters was obtained from Tang et al.^22^ (GSM1872886). Following the methods of the original paper, we removed from the consideration CTCF-PET clusters that had less than four interactions. Then we binned the resulting CTCF-PET cluster interaction map using a 10 kb window. The resulting interaction coordinates of 10 kb windows, we considered as CTCF-mediated loops. The data for CTCF-HiChIP in .hic format was obtained from Mumbach et al.^26^ (GSE115524). In accordance with the original paper, we annotated CTCF-mediated interactions using HiCCUPS with the following parameters: -m 500 -r 5000, 10,000 -f 0.1,0.1 -p 4,2 -i 7,5 -d 20,000, 20,000.

### CTCF, ATAC-seq, and H3K27AC peaks

Data for GM12878 cells were obtained from the ENCODE (ENCFF410XEP, ENCFF411MHX, ENCFF710VEH). Coordinates of ATAC-seq peaks in hg38 have been translated to hg19 using the LiftOver utility (https://genome.ucsc.edu/cgi-bin/hgLiftOver). The base of the loop was considered to contain a peak if it contained at least one peak from the corresponding mark.

### Identification of enhancer-promoter interactions

To identify enhancer-promoter interactions, we used the LASCA pipeline on GM12878 Hi-C data at 25 kb resolution without filters and clustering. Next, we selected significant contacts at scales up to 25 kb. We defined promoters as regions from the gene transcription start site (TSS) to 1 kb upstream of that TSS. We intersected these promoters and significant contacts and left only those contacts for which one of the anchors fell inside the promoter region; another anchor, therefore, was assigned as an enhancer.

## Supplementary Information

Supplementary Information.

## Data Availability

LASCA, the protocol, and suite of scripts, are publicly available at https://github.com/ArtemLuzhin/LASCA_pipeline. Hi-C datasets for *C. elegans* (GSE132640), and GM12878 (GSE63525) and CH12-LX cell lines (GSE63525) were downloaded from NCBI GEO. The Hi-C dataset for *S. cerevisiae* (PRJNA427106) was downloaded from NCBI Bioproject. ChIP-seq and DNAse I sensitivity datasets for GM12878 (ENCFF312KXX, ENCFF158GBQ, ENCFF167NBF, ENCFF180LKW, ENCFF682WPF, ENCFF776OVW), and CH12-LX (ENCFF025UEN) were downloaded from ENCODE Project. ChIP-seq for *S. cerevisiae* (GSM1712307) and *C. elegans* (GSM3680196) were downloaded from NCBI GEO.
