# Supplementary material for: LASCA: loop and significant contact annotation pipeline
Source: Sci Rep. 2021 Mar 18;11:6361. doi: 10.1038/s41598-021-85970-4 (PMC7973524; doi:10.1038/s41598-021-85970-4)
Supplement: Supplementary file 1 — Supplementary Information. [file 41598_2021_85970_MOESM1_ESM.pdf]

SUPPLEMENTARY INFORMATION

LASCA: loop and significant contact annotation pipeline

Artem V. Luzhin, Arkadiy K. Golov, Alexey A. Gavrilov, Artem K. Velichko, Sergey V. Ulianov, Sergey V. Razin & Omar L. Kantidze

Scientific Reports 2021

This file contains:
Supplementary Figures S1-S3

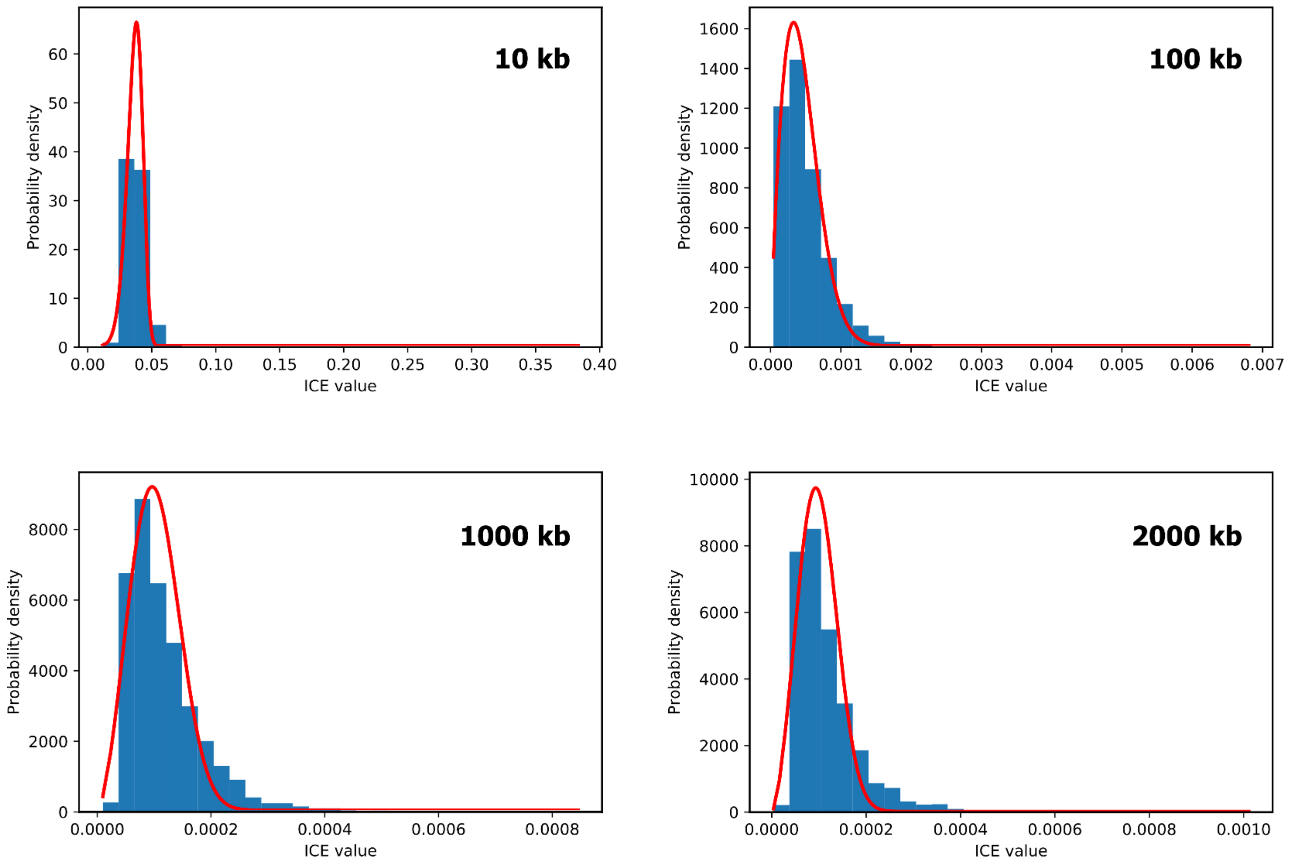

Supplementary Figure S1. Weibull distributions fitted in distributions of Hi-C interaction frequencies at three different equidistant matrix diagonals (10, 100, 1000, and 2000 kb).

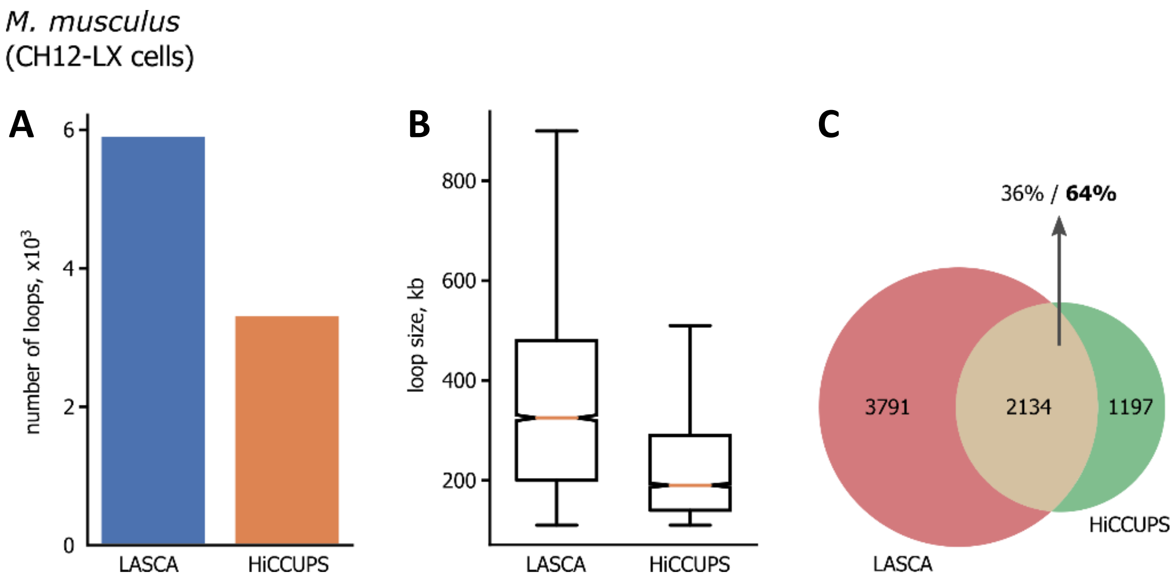

Supplementary Figure S2. Comparison of LASCA pipeline with HiCCUPS. (A) Number of loops identified by LASCA and HiCCUPS in mouse (CH12-LX) Hi-C datasets. (B) Sizes of loops identified by LASCA and HiCCUPS in mouse (CH12-LX) Hi-C datasets. Horizontal lines represent the median; upper and lower ends of boxplot show the upper and lower quartiles, the whiskers indicate the upper and lower fences. (C) The agreement between a LASCA and HiCCUPS loops is shown as Venn diagrams. The overlap between the two loop sets is shown in yellow, and the percentages of overlap with respect to each set are reported separately.

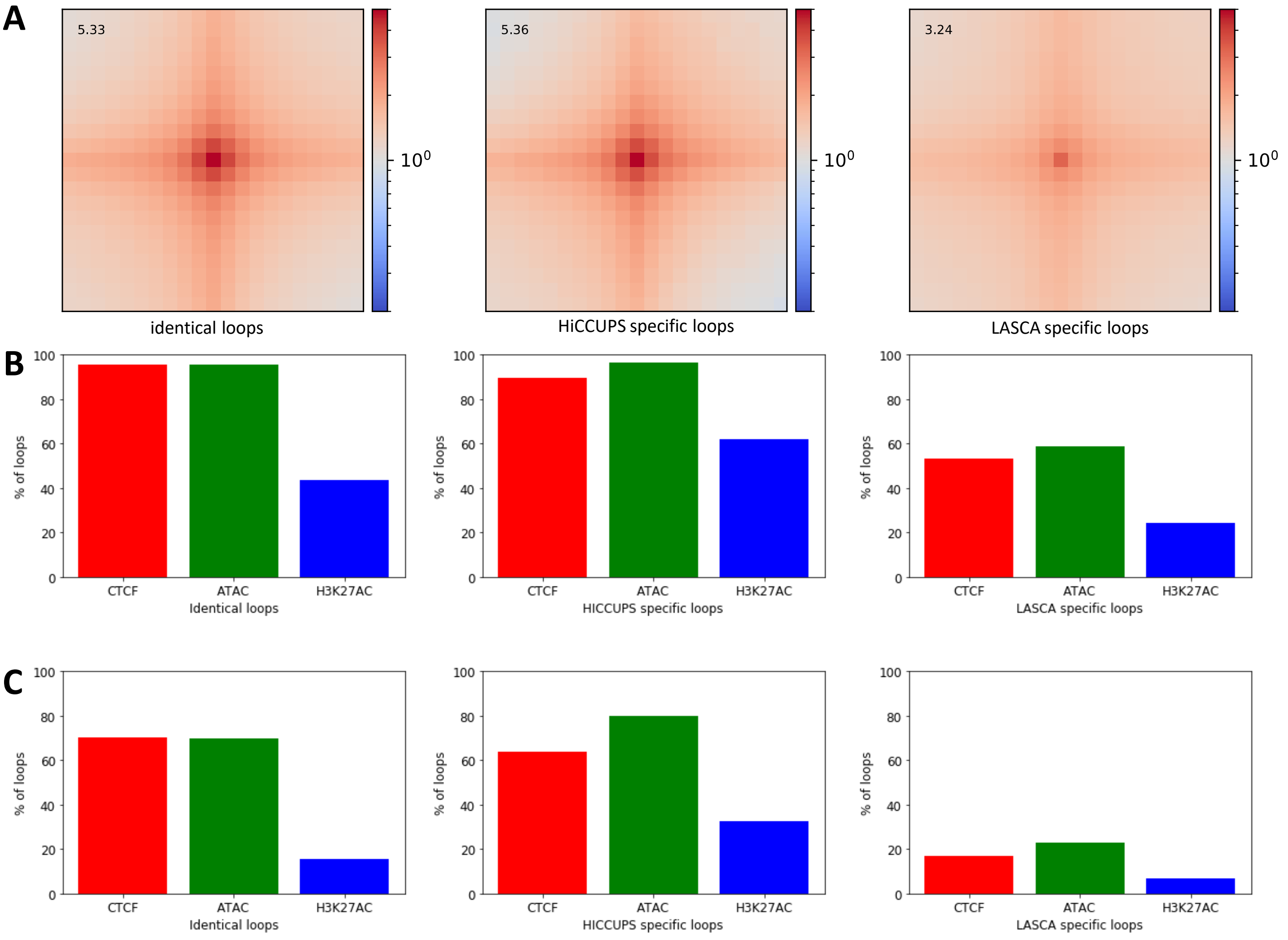

Supplementary Figure S3. Independent analyses of i) loops identified by both LASCA and HiCCUPS, ii) HiCCUPS-specific loops, and iii) LASCA-specific loops (GM12878 cell line). (A) Average loops identified by LASCA and grouped as described above. A number in the upper left corner shows the enrichment of contacts inside the loop pixel over the background. (B-C) Percent of LASCA identified loops that contain CTCF binding sites (red), ATAC-seq peaks (green) or histone H3K27Ac mark (blue) at no less than one base point (B) or at both base points.
